# Risk Distribution of Human Infections with Avian Influenza H7N9 and H5N1 virus in China

**DOI:** 10.1038/srep18610

**Published:** 2015-12-22

**Authors:** Xin-Lou Li, Yang Yang, Ye Sun, Wan-Jun Chen, Ruo-Xi Sun, Kun Liu, Mai-Juan Ma, Song Liang, Hong-Wu Yao, Gregory C. Gray, Li-Qun Fang, Wu-Chun Cao

**Affiliations:** 1 The State Key Laboratory of Pathogen and Biosecurity, Beijing Institute of Microbiology and Epidemiology, Beijing 100071, P. R. China; 2 Department of Biostatistics, College of Public Health and Health Professions & Emerging Pathogens Institute, University of Florida, 100188, Florida, USA; 3 Department of Environmental and Global Health, College of Public Health and Health Professions, and Emerging Pathogens Institute, University of Florida, 100188, Florid, USA; 4 Division of Infectious Diseases, Global Health Institute, &Nicholas School of the Environment, Duke University, 27710, Durham, NC, USA

## Abstract

It has been documented that the epidemiological characteristics of human infections with H7N9 differ significantly between H5N1. However, potential factors that may explain the different spatial distributions remain unexplored. We use boosted regression tree (BRT) models to explore the association of agro-ecological, environmental and meteorological variables with the occurrence of human cases of H7N9 and H5N1, and map the probabilities of occurrence of human cases. Live poultry markets, density of human, coverage of built-up land, relative humidity and precipitation were significant predictors for both. In addition, density of poultry, coverage of shrub and temperature played important roles for human H7N9 infection, whereas human H5N1 infection was associated with coverage of forest and water body. Based on the risks and distribution of ecological characteristics which may facilitate the circulation of the two viruses, we found Yangtze River Delta and Pearl River Delta, along with a few spots on the southeast coastline, to be the high risk areas for H7N9 and H5N1. Additional, H5N1 risk spots were identified in eastern Sichuan and southern Yunnan Provinces. Surveillance of the two viruses needs to be enhanced in these high risk areas to reduce the risk of future epidemics of avian influenza in China.

Global concerns regarding the possibility of an avian influenza pandemic have been accumulating since human infection with avian influenza A (H5N1) was first reported in 1997. By the end of 2014, this virus has caused 47 laboratory-confirmed human infections with 34 deaths in mainland China^1^. The situation became more worrisome with the recent outbreaks of the novel influenza A (H7N9) in China^2^. By the end of 2014, a total of 478 confirmed human H7N9 cases with 208 deaths have been reported in 13 provinces and two municipalities in mainland China^3^. In addition, recently reported human infections with novel reassortant of avian influenza A H10N8 virus and H5N6 virus, as well as a recent outbreak of H5N2 virus in poultry in China, further highlight the ongoing reassortment among avian influenza viruses^4^,^5^,^6^. Thus far, there has been no evidence for sustainable person-to-person transmission of H5N1, H7N9, H10N8, H5N6 or H5N2. While both are of avian origin, H7N9 and H5N1 exhibit prominent differences in epidemiological, virological and immunological characteristics^7^,^8^,^9^. For example, H7N9 is low pathogenic in birds, whereas H5N1 is highly pathogenic^10^. The fatality risk among hospitalized H5N1 human infection was higher than that among hospitalized H7N9 infections^7^. Studies have also reported that hospitalized H7N9 cases were elder than hospitalized H5N1 cases^7^,^8^,^9^. Although most cases in urban areas were men for both viruses, the proportion of males among all cases in rural areas was about 60% for H7N9 but was only about 30% for H5N1^7^. Human infections of H5N1 were scattered spatially, whereas human infections of H7N9 were more clustered^7^,^8^. Despite the differences in various aspects, local co-circulation and even co-infection of H5N1 and H7N1 are possible. Previous studies have identified areas in which human infections with H7N9 and those with H5N1 were both reported^8^,^9^. Serological studies have suggested co-infections of H7N9 and H5N1 among herons in southern China, indicating the potential of reassortment between the two viruses^11^.

It remains unclear what factors have contributed to the heterogeneity in the spatial distribution of human infections between H7N9 and H5N1. Identification of high risk areas for human infections with these viruses, where surveillance should be enhanced and intervention programs should be placed and would be crucial to reduce the risk of epidemics and viral reassortment. Our study aims to quantify the relative contributions of agro-ecological, environmental and meteorological factors to human H7N9 and H5N1 infections in China, and to map the high risk areas for the occurrence and concurrence of the two viruses.

## Results

**Figure 1:**
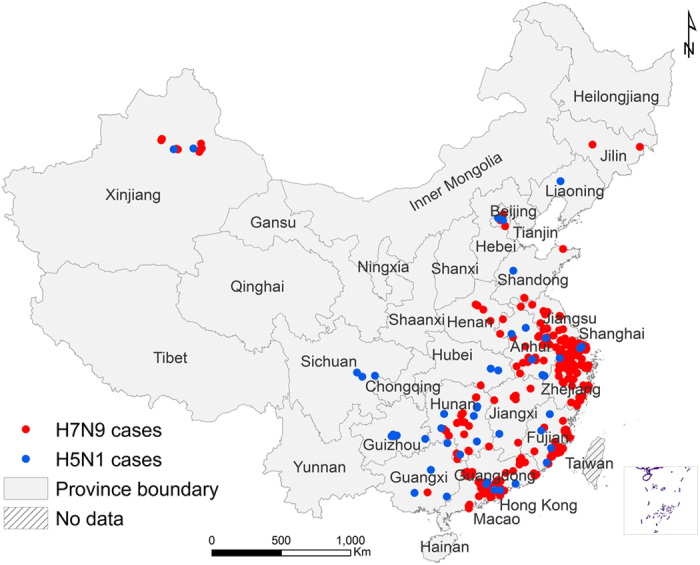
Spatial distribution of human infections with avian influenza A (H7N9) and with A (H5N1) in mainland China from 2003 to 2014. Red (blue) dots indicate the locations of human H7N9 (H5N1) cases. The map was created in ArcGIS 9.3 software (ESRI Inc., Redlands, CA, USA) (http://www.esri.com/).

By the end of 2014, a total of 478 avian influenza A (H7N9) and 47 A (H5N1) laboratory-confirmed human cases have been reported in mainland China. Figure 1 displays the spatial distribution of human H7N9 cases in 210 counties of 13 provinces and two municipalities in eastern China, and the distribution of human H5N1cases in 45 counties of 14 provinces and two municipalities. Human cases with H7N9 infection were shown to be more spatially clustered than those with H5N1 infection. The epidemics of the two pathogens both showed strong seasonality. The H7N9 epidemics usually peak during January ─ April (85.1% of all cases), slightly later than the peak season of the H5N1 outbreaks during November ─ February (77.8% of all cases) (Supplementary Fig. S1 online). Based on the boosted regression tree (BRT) models, live poultry markets, the density of human population, the percentage coverage of built-up land, relative humidity and precipitation were found to be significant contributors to the spatial distributions of both viruses. In particular, precipitation has mean weights >20% for both viruses. However, each virus also has its own ecological drivers for human infection. The density of poultry, the percentage coverage of shrub and temperature played important roles in the occurrence of human infection with H7N9 (BRT mean weights are 5.27%, 5.11%, and 5.17%, respectively), while the presence of human H5N1 infection was significantly associated with the percentage coverage of forest and the percentage coverage of water body (BRT mean weights are 5.51% and 5.68% respectively) (Table 1). The distances to the nearest freeway or national highway, the percentage coverage of cropland and the percentage coverage of wetland were not found to be substantially associated with human infections with either H7N9 or H5N1, all of which had BRT relative contributions <2.0% and were thus excluded from the final models.

For both viruses, a higher risk of human infection was associated with increases in the number of live poultry markets, the density of human population, the percentage coverage of built-up land, and precipitation (Supplementary Fig. S2 and S3 online). Although the two viruses share relative humidity as a common risk factor, the effect patterns differ between them. A higher relative humidity (after 70%) was associated with a higher risk of H5N1 infection (Supplementary Fig. S3 (F) online). In contrast, the effect was not monotonic for H7N9. The risk peaks at a relative humidity of 75% and falls quickly after that (Supplementary Fig. S2 (G) online). An elevated risk of H7N9 infection was associated with a higher density of poultry and lower shrub coverage. The effect of temperature on the risk of H7N9 infection also appears to be non-monotone, with the peak risk reached at 22 °C (Supplementary Fig. S2 (F) online). A higher forest coverage or a higher coverage of water body implied a higher risk of H5N1 infection, but the effect sizes were quite small (Supplementary Fig. S3 (C) and (E) online).

To determine the discriminatory ability of the BRT models, the receiver-operating characteristic (ROC) curves were produced and areas under the curve (AUC) were calculated and shown (Supplementary Fig. S4 online). The average discriminatory ability of the BRT models over 50 resamples was 96.21% (95% CI: 95.01%−97.41%) for H7N9 and 87.76% (95% CI: 82.32%−93.20%) for H5N1, respectively (Supplementary Fig. S4 online), indicating a decent predictive power of our models for the risks of human infection with H7N9 and with H5N1.

**Figure 2:**
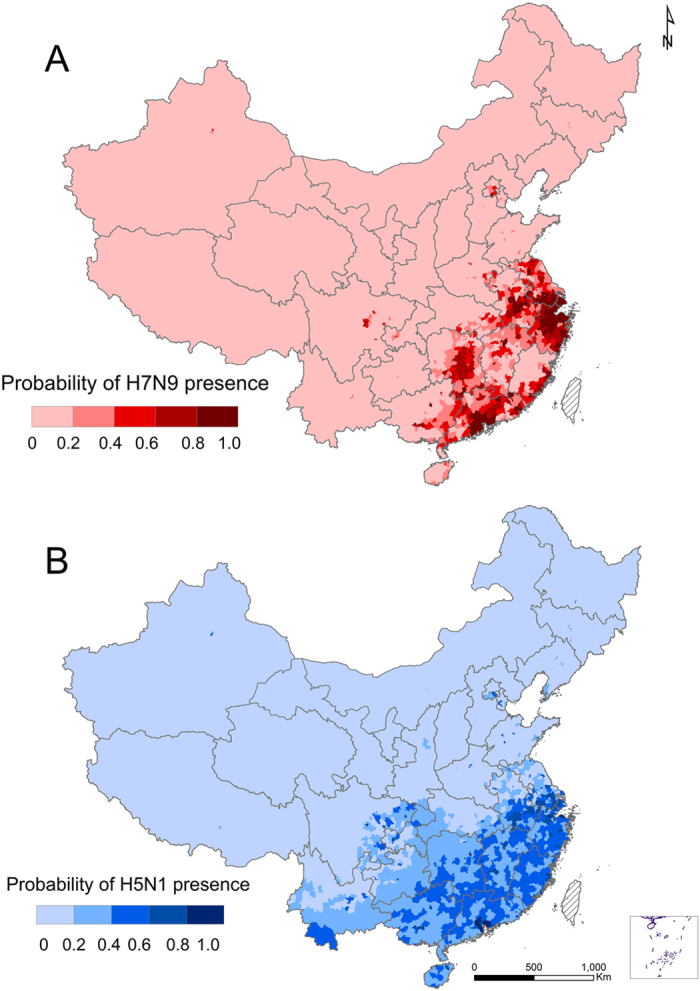
Predictive risk maps of probability of occurrence of human infections with H7N9 and with H5N1 in mainland China. (**A**) Human infections with H7N9, darker red indicating a higher risk, (**B**) Human infections with H5N1, darker blue indicating a higher risk. The map was created in ArcGIS 9.3 software (ESRI Inc., Redlands, CA, USA) (http://www.esri.com/).

The spatial distribution of the model-fitted probabilities of occurrence of human infections was shown in Fig. 2A for H7N9 and in Fig. 2B for H5N1. The distributions of high risk areas were similar between the two viruses, with the majority of these areas located in southeastern China, extending from the Pearl River delta near Guangzhou to the Yangtze River delta near Shanghai and covering most areas of Jiangsu, Zhejiang, Shanghai, Anhui, Fujian, Hunan, Jiangxi, Guangdong and Guangxi Provinces. Additional hot spots for human infections with H5N1 were found in the eastern region of Sichuan Province, Chongqing metropolis, the southern region of Yunnan Province, and Hainan Province (Fig. 2B). The high risk areas for H7N9 were more spatially clustered than those for H5N1. Restricting the analysis to the main epidemic seasons rather than using the data from all seasons showed little change in the selection of important predictors, though the relative contributions of the predictors did show a little variation (supplementary Table S2 online).

**Figure 3:**
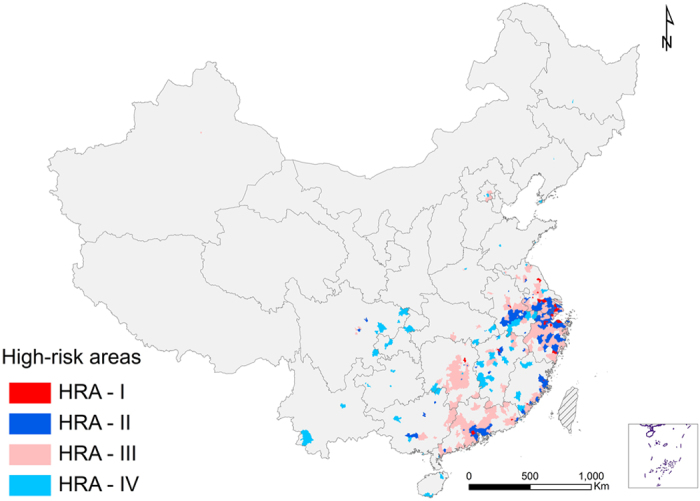
Model-predicted high risk areas (HRAs) for the occurrence of human infections with H7N9 and with H5N1 in mainland China. HAR-I (red) represents counties with predicted probabilities of occurrence of human infection >50% for both H7N9 and H5N1, above-average densities of swine and poultry, and below-average distance from their centroid to one of the nearest important bird areas; HAR-II (lazuli) represents counties with predicted probabilities of occurrence of human infection >50% for both H7N9 and H5N1 but not in HRA-I; HAR-III (rose) represents counties with a predicted probability of occurrence of human H7N9 virus infection >50% but no in HRA-I and HRA-II; HAR-IV (blue) represents counties with a predicted probability of occurrence of human H5N1 virus infection >50% but not in HRA-I and HRA-II. The map was created in ArcGIS 9.3 software (ESRI Inc., Redlands, CA, USA) (http://www.esri.com/).

Figure 3 demonstrates the distribution of the four types of high risk areas (HRAs), which are defined by the classification of counties according to the model-predicted risk, the densities of swine and poultry, and the distance to the nearest important bird area. HRA-I and HRA-II were mainly clustered in the Yangtze River Delta in eastern China (Shanghai, Jiangsu, Anhui, and Zhejiang provinces) and Pearl River Delta (part of Guangdong Province surrounding Guangzhou), along with a few spots on the southeast coastline in Fujian Province. Sporadic HRA-I and -II were also seen in Hunan, Jiangxi and Sichuan Provinces. These areas presented the highest risk for the concurrence of human H7N9 and H5N1infections. They could serve as hotbeds for genetic reassortment among avian influenza viruses including H7N9 and H5N1. The distribution of HRA-III counties is similar to that of HRA-I and HRA-II, with a wider spatial range in these provinces. These counties should be monitored for increasing risk of occurrence of human H7N9 infections. HRA-IV, which poses higher risks of human H5N1 infection, extended to a wider range than the other three HRA types and covered the whole southern China. The total area, the cumulative incidences of human H7N9 and H5N1 infections, the numbers of poultry, swine and live poultry markets, and the number of important bird areas within 124 km (the average distance from all the counties to their nearest important bird areas) were summarized for each HRA type in Table 2. Although HRA-I and HRA-II occupy less than 30% of the area of HRA-III and HRA-IV, H7N9 incidences in these counties were twice that of HRA-III, and H5N1 incidences in these counties were comparable to that of HRA-IV. In particular, HRA-I and HRA-II also had more live poultry markets than HRA-III and HRA-IV.

## Discussion

Differences in individual-level characteristics such as age, sex and occupation between human H7N9 and H5N1 infections have been previously reported^7^,^8^,^9^. Our study is the first to examine the difference in the spatial patterns and ecological risk factors between human H7N9 and H5N1 infections. It is interesting that, while human infections with H7N9 and H5N1 share similar exposure-related (live poultry markets and the density of human population) and environmental risk factors (the coverage of built-up land, relative humidity and precipitation), each virus possesses its own ecological risk factors. Human infection with H7N9 is affected by the density of poultry, the coverage of shrub and temperature, whereas the risk of H5N1 is modified by the coverage of forest and the coverage of water body. The contribution of the density of poultry on the presence of H7N9 may be explained by potential endemics of the virus among the poultry population due to its low pathogenicity in poultry, which is also supported by the fact that positive tests for H7N9 in poultry and related environments were spatially scattered over the nation, according to the national active virological surveillance conducted by the Ministry of Agriculture of China. Conversely, the relatively weak association between the density of poultry and the risk of human H5N1 infection implies the lack of sustainable long term transmission among domestic poultry, likely a consequence of the high pathogenicity of the virus in poultry. The significant contribution of the coverage of forest and water body to the risk of human H5N1 infection may imply a transmission route from wild birds to domestic poultry and then to human. Wild birds, in particular migratory waterfowl, are known to be natural reservoirs for H5N1 virus, and the virus can be low pathogenic in these birds^12^,^13^. The forests and waterbodies are natural habitat for waterfowl, and water bodies also provide mixing venue for both wild and domestic waterfowl^14^.

Live poultry markets have been considered as the reservoir and amplifier for avian influenza viruses, as the animal hosts are kept in highly crowded conditions that facilitate frequent transmission and probable genetic reassortment^2^,^10^,^12^,^15^,^16^,^17^,^18^,^19^,^20^. Live poultry markets had a higher contribution to the occurrence of human H5N1 infection than to the occurrence of human H7N9 infection in our model. This difference might result from the wider range of circulation of H7N9 compared to H5N1. Because of the high pathogenicity of H5N1 in poultry and the nationwide vaccination campaigns in poultry starting 2006, large live poultry markets might have become the main venue for human exposure to H5N1 virus, while the low pathogenic H7N9 has spread to small poultry markets or general markets with poultry stalls^7^,^14^. Our data might have only included live poultry markets of large scale. In addition, the difference could also be partially explained by the different transmissibility from poultry to person between H7N9 and H5N1 viruses given the same level of contact in live poultry markets, which warrants future investigation^7^.

The important role of the density of human population for both H7N9 and H5N1 is not surprising. A higher density of human population is usually associated with a higher volume of poultry trading and consumption^21^. The coverage of built-up land is also positively related to poultry trading and might have increased the chance for human exposure. Relative humidity and precipitation played significant roles in human infections for both viruses, but temperature only affected the occurrence of human H7N9 infections, consistent with our previous studies for the two viruses in early years^15^,^22^.

This study has some limitations. Our analyses were based on laboratory-confirmed cases of human H7N9 and H5N1 infections, and are therefore subject to reporting bias, e.g., information about subclinical or mild infections with the two viruses was not captured due to the nature of the two diseases and the surveillance systems, especially in some less developed areas of China^17^. In addition, the pathogenicity in human possibly differs between the two viruses, and the impact of such difference on the risk assessment is worth further exploration. It is also possible that some symptomatic infections were not detected due to inadequate surveillance, especially in less developed areas of China^21^. Sero-surveillance studies may help alleviate this problem, but such data are either scarce or difficult to obtain. Secondly, the small number of human H5N1 infections reported in China has limited the discriminatory ability of the BRT models for H5N1, as evidenced by the lower AUCs and larger standard deviations of the BRT weights compared to those for H7N9. The sparsity of human H5N1 infection has also made its seasonality difficult to define. Finally, due to data availability, our analysis did not consider animal infections with the two viruses.

Given the more and more frequent occurrence of novel reassortment avian influenza viruses, targeted surveillance efforts and preventive interventions should be actively implemented in the high risk areas we identified, particularly in the HRA-I counties where the high probabilities of concurrence of human infections with H7N9 and with H5N1 viruses are coupled with high densities of poultry and swine and proximity to important birds areas, a suitable ecological background for the co-circulation of H7N9 and H5N1 viruses. Active surveillance of avian viruses in both human and animal reservoirs including poultry, swine and wild birds in the high risk areas would be valuable to reduce the risk of human infections and the risk of viral reassortment. We also emphasize on the need for data collection, sharing and analysis to be conducted in a more open and cooperative fashion and under a one-health framework among relevant parties, such as public health agencies, animal surveillance agencies and non-governmental research institutes.

## Materials and Methods

### Data on human cases with H7N9 and H5N1 virus infections

The data regarding all laboratory-confirmed cases infected with avian influenza A (H7N9) and (H5N1) in China from 2003 to 2014 were collected from the World Health Organization^23^, and the consistency with the data from Health Map (http://www.healthmap.org/zh/) and Flu Trackers (https://flutrackers.com/forum/) was checked to ensure data quality and reliability. The date of onset, age, gender and location of each confirmed patient was extracted and used in this study. The definitions of H5N1 and H7N9 human infections have been described as previously^15^,^24^. As in previous studies^14^,^15^,^22^, each confirmed case was geo-referenced and linked to a digital map of China according to each patient’s residence at the time of symptom onset using GIS technologies. Monthly cumulative numbers of cases based on their symptom onset dates were plotted over time to display the temporal trend of the epidemics caused by the two viruses.

### Mapping the risk distribution of human infections with H7N9 and H5N1 virus

To identify risk factors for the spatial distribution of human infections with H7N9 and H5N1, the following county-level agro-ecological, environmental and meteorological data from 2003 to 2014 were collected (Supplementary Table S1 online): the density of human population, the density of poultry, the number of live poultry markets, land cover variables such as the percentage coverage of forest, shrub, grassland, cropland, built-up land, wetland, and water bodies, and meteorological variables including temperature, relative humidity and precipitation. As a proxy for both poultry-trading level and the size of susceptible human hosts, the density of human population has been found to influence the risk of human cases of avian influenza^15^,^16^,^21^,^22^,^25^,^26^,^27^,^28^. The poultry-related variables represent the exposure level of human hosts to the potential sources of infection. The land-cover variables are potential predictors for their association with habitats and food sources for wild birds and domestic poultry. For example, water bodies constitute the natural habitat for waterfowl, and croplands and waterbodies create a mixing venue between free-ranging domestic poultry and wild waterfowl, and their mixing may facilitate cross-species avian influenza transmission^14^,^15^,^16^,^21^,^22^,^27^,^29^. Meteorological factors have been shown to be drivers for human infection with H5N1virus in Egypt and Indonesia^30^ and for the first epidemic wave of human infections with H7N9 virus in China^15^.

The density of human population at the county level was obtained from the National Bureau of Statistics of China. The density of poultry and swine were available from the Food and Agriculture Organization of the United Nations^31^. The geographic locations of live poultry markets in 2012 in mainland China were obtained from AutoNavi (www.autonavi.com), which is a platform for web mapping and location-based services provider in China. The AutoNavi platform collected Point of Interest (POI) cross the mainland China, which includes the locations for live poultry markets. It is possible that small or mobile live poultry markets are not included in the AutoNavi database. Land-cover data at the 1 km × 1 km resolution in 2005 were obtained from the Data Sharing Infrastructure of Earth System Science (www.geodata.cn). Monthly meteorological variables including cumulative precipitation, average relative humidity and average temperature during the study period were obtained from the Chinese Academy of Meteorological Sciences (www.cams.cma.gov.cn).

To explore the contribution of the ecological variables to the occurrence of human H7N9 or H5N1 infections and to map the corresponding risk distributions, boosted regression tree (BRT) models were constructed at the county level based on a “case-control” design (explained below). BRT models are efficient for predicting distributions of organisms while accounting for non-linear covariate-response relationships and interactions between covariates, and therefore have been widely used to identify risk determiners for various zoonotic diseases^16^,^21^,^32^. In this study, influenza A (H7N9) and (H5N1) were modeled separately. For each model, all counties with laboratory-confirmed cases were regarded as “cases”, and five-fold “controls” were randomly selected from all counties without reported cases, where the case-control ratio is based on previous studies^14^,^15^,^21^,^22^. Building a BRT model is a stage-wise process. At each stage, the optimal tree is found to explain the residuals of the model from the previous stage and is linearly combined with the existing trees. A bootstrap data set was drawn for tree-building at each stage to provide robust estimation of the model parameters. A tree complexity of 5, a learning rate of 0.005 and a bag fraction of 75% were used to identify the optimal tree for each bootstrap data. The relative weight for each variable was estimated from the identified trees and served as an indicator for the relative importance of that variable in predicting the outcome. Variables that had a low contribution to the occurrence of the disease (weight <2%) were excluded from the final model. Details on the modeling procedure were described in one of our previous studies^15^. As control counties were randomly selected, to ensure robust inference, the above model-building procedure was repeated over 50 resampled datasets (only controls were resampled). We report the mean values and standard deviations of the weights over the 50 resampled datasets. Risk maps for the presence of human infections with avian influenza A (H7N9) or A (H5N1) virus were created based on the predicted probabilities over the 50 resamples. The predictive power of these models was evaluated using ROC curves and the area under the curve (AUC). In addition, to reduce the impact of sparse data, sensitivity BRT analyses were conducted using the data restricted to the main epidemic seasons for the two viruses (85.1% of all human H7N9 cases during January — April and 77.8% of all human H5N1 cases during November — February).

### Risk assessment

To identify the areas with elevated probability of genetic reassortment between H7N9 and H5N1, we considered not only the model-predicted risks of human infections with the two viruses but also the density of poultry, density of swine and the distance to important bird habitat areas. Counties with high densities of poultry and swine are potential breeding ground for the H7N9 and H5N1 viruses to circulate, and wild birds are known to be able to carry avian influenza viruses over long distances and to introduce the viruses into new areas during migration^33^. Although the avian H7N9 virus has not been detected in swine or migratory birds up to now^9^,^34^, it is known to infect tree sparrows^35^. The data regarding important habitat areas along the migratory routes of wild birds across China were collected from the Directory of Important Bird Areas in China (mainland): Key Sites for Conservation by Bird Life International in 2009 (http://www.chinabirdnet.org/iba_inventory.html). Important habitat areas were categorized into four types in the original data: (1) A1: Areas for globally threatened species^36^; (2) A2: Areas for restricted-range species^37^,^38^; (3) A3: Areas for biome-restricted assemblages^39^; (4) A4: Areas for globally important congregations^40^. In this study, a total of 191 A4 areas were included in the analyses to represent the important sites for migration waterfowl.

We classified all counties >50% model-predicted risks of human H5N1 or H7N9 infection into four types of high-risk areas (HRAs)^41^: (1) HRA-I, counties with predicted probabilities of occurrence of human infection >50% for both H7N9 and H5N1, above-average densities of swine and poultry, and a below-average distance from the centroid to the nearest important bird area; (2) HRA-II, counties with predicted probabilities of occurrence of human infection >50% for both H7N9 and H5N1 but not in HRA-I; (3) HRA-III, counties with a predicted probability of occurrence of human H7N9 infection >50% but not in HRA-I and HRA-II; (4) HRA-IV, counties with a predicted probability of occurrence of human H5N1 infection >50% but not in HRA-I and HRA-II. The distribution of the four types of HRAs was shown on a map, and we summarized the total area, the cumulative incidences of human H7N9 and H5N1 infections, and the numbers of poultry, swine, and live poultry markets, as well as the number of important bird areas with 124 km (the average distance from all counties to their nearest important bird areas) for each of the four types of HRAs.

## Additional Information

**How to cite this article**: Li, X.-L. *et al.* Risk Distribution of Human Infections with Avian Influenza H7N9 and H5N1 virus in China. *Sci. Rep.* **5**, 18610; doi: 10.1038/srep18610 (2015).

## Supplementary Material

Supplementary Information

## Figures and Tables

**Table 1:** Results of the boosted regression trees model applied to human H7N9 and H5N1 infections reported in China as of 2014.

Variables†	Relative contribution			
	Human H7N9 infection		Human H5N1 infection	
	Mean (%)	Sd	Mean (%)	Sd
Number of live poultry markets	7.58	1.35	18.92	6.10
Density of poultry	5.27	1.03	2.19	1.36
Density of human population	6.82	1.76	9.42	3.82
Freeway	NS	—	2.68	1.37
National highway	NS	—	3.04	2.18
Percentage coverage of forest	3.55	0.83	5.51	2.13
Percentage coverage of shrub	5.11	1.43	NS	—
Percentage coverage of grassland	3.39	0.70	3.48	1.94
Percentage coverage of croplands	NS	—	3.19	2.01
Percentage coverage of built-up land	17.81	2.65	8.35	4.42
Percentage coverage of water body	3.82	0.89	5.68	3.27
Percentage coverage of wetland	2.41	0.68	NS	—
Temperature	5.17	0.84	3.24	1.64
Relative humidity	16.98	1.99	9.50	4.68
Precipitation	22.10	1.88	24.80	4.85

“NS”: These variables were excluded from the final model due to small BRT weights (<2.0%).

^†^Variables with mean weights ≥ 5% were considered as significant contributors to the occurrence of human infections.

**Table 2:** Total area, cumulative incidences of human infections with H7N9 and with H5N1, the numbers of poultry, swine, live poultry markets, and important bird areas by the type of HRA.

Type of high risk areas	Total area (1,000 km^2^)	Cumulative incidence of human infection		No. of poultry (millions)	No. of swine (millions)	No. of live poultry markets	No. of important bird areas
		H7N9 (1/100,000)	H5N1 (1/100,000)				
HRA-I^a^	9.5	0.217	0.011	21.3	1.6	494	40
HRA-II^b^	83.2	0.276	0.016	83.4	6.8	1351	49
HRA-III^c^	249.0	0.105	0.003	239.8	25.8	726	69
HRA-IV^d^	82.6	0.002	0.012	48.9	8.0	653	85

^a^HRA-I, counties with predicted probabilities of occurrence of human infection >50% for both H7N9 and H5N1, above-average densities of swine and poultry, and below-average distance from their centroid to one of the nearest important bird areas.

^b^HRA-II, counties with predicted probabilities of occurrence of human infection >50% for both H7N9 and H5N1 but not in HRA-I.

^c^HRA-III, counties with a predicted probability of occurrence of human H7N9 virus infection >50% but no in HRA-I and HRA-II.

^d^HRA-IV, counties with a predicted probability of occurrence of human H5N1 virus infection >50% but not in HRA-I and HRA-II.
