# Supplementary material for: Risk Distribution of Human Infections with Avian Influenza H7N9 and H5N1 virus in China
Source: Sci Rep. 2015 Dec 22;5:18610. doi: 10.1038/srep18610 (PMC4686887; doi:10.1038/srep18610)

**Supplementary Information: Risk Distribution of Human Infections with Avian Influenza H7N9 and H5N1 virus in China**

Xin-Lou Li^1^, Yang Yang^2^, Ye Sun^1^, Wan-Jun Chen^1^, Ruo-Xi Sun^1^, Kun Liu^1^, Mai-Juan Ma^1^, Song Liang^3^, Hong-Wu Yao^1^, Gregory C. Gray^4^, Li-Qun Fang^1*^, Wu-Chun Cao^1*^

1. The State Key Laboratory of Pathogen and Biosecurity, Beijing Institute of Microbiology and Epidemiology, Beijing 100071, P. R. China,
2. Department of Biostatistics, College of Public Health and Health Professions & Emerging Pathogens Institute, University of Florida, 100188, Florida,
3. Department of Environmental and Global Health, College of Public Health and Health Professions, and Emerging Pathogens Institute, University of Florida, 100188, Florid, USA
4. Division of Infectious Diseases, Global Health Institute, &Nicholas School of the Environment, Duke University, Durham, NC, USA

* Correspondence to: Wu-Chun Cao and Li-Qun Fang, the State Key Laboratory of Pathogen and Biosecurity, Beijing Institute of Microbiology and Epidemiology, Beijing 100071, P. R. China, caowc@bmi.ac.cn and fang_lq@163.com

**Key words:** avian influenza A (H7N9); avian influenza A (H5N1); risk factors; risk assessment; China;

**Short title:** Risk distribution of H7N9 and H5N1 in China

**1. Table S1**

**2. Table S2**

**2. Figure S1**

**3. Figure S2**

**4. Figure S3**

**5. Figure S4**

**Table S1. Description of potential risk factors used in the BRT** analyses.

| Abbr. | Variables | Description (Unit) | Type |
| --- | --- | --- | --- |
| PM | Live poultry markets | Number of live poultry markets for each county (market per county) | Continuous |
| PD_1 | Density of poultry | Poultry density for each county (feature per km^2^) | Continuous |
| PD_2 | Density of human population | Population density for each county (1,000 persons per county) | Continuous |
| DF | Freeway | Distance from centroid of each county to the nearest freeway (km) | Continuous |
| DNH | National highway | Distance from centroid of each county to the nearest national highway (km) | Continuous |
| FS | Forest | Percentage coverage of forest for each county (1%) | Continuous |
| SH | Shrub | Percentage coverage of shrub for each county (1%) | Continuous |
| GL | Grassland | Percentage coverage of grassland for each county (1%) | Continuous |
| CL | Cropland | Percentage coverage of cropland for each county (1%) | Continuous |
| BU | Built-up land | Percentage coverage of built-up land for each county (1%) | Continuous |
| WB | Water body | Percentage coverage of waterbody for each county (1%) | Continuous |
| WL | Wetland | Percentage coverage of wetland for each county (1%) | Continuous |
| TP | Temperature | Average temperature during the study period for each county (℃) | Continuous |
| RH | Relative humidity | Average relative humidity during the study period for each county (1%) | Continuous |
| PRE | Precipitation | Annul precipitation during the study period for each county(mm) | Continuous |

**Table S2** Results of the boosted regression trees model when the analysis is restricted to the peak months of human H7N9 and H5N1 infections in China as of 2014.

|  | Relative contribution | | | |
| --- | --- | --- | --- | --- |
| Variables† | Human H7N9 infection | | Human H5N1 infection | |
|  | Mean (%) | Sd | Mean (%) | Sd |
| Number of live poultry markets | 5.17 | 1.07 | 21.27 | 6.02 |
| Density of poultry | 5.69 | 1.19 | NS | - |
| Density of human population | 5.12 | 2.04 | 8.91 | 4.24 |
| Freeway | NS | - | 3.39 | 1.50 |
| National highway | 2.58 | 0.57 | 2.48 | 1.81 |
| Percentage coverage of forest | 3.48 | 0.61 | 7.25 | 3.43 |
| Percentage coverage of shrub | 6.03 | 1.87 | NS | - |
| Percentage coverage of grassland | 3.70 | 1.11 | 4.20 | 2.31 |
| Percentage coverage of croplands | NS | - | 8.61 | 4.01 |
| Percentage coverage of built-up land | 11.73 | 2.05 | 3.23 | 2.14 |
| Percentage coverage of water body | 3.30 | 0.64 | 5.89 | 2.96 |
| Percentage coverage of wetland | 2.57 | 0.68 | NS | - |
| Temperature | 4.79 | 0.78 | 6.41 | 2.69 |
| Relative humidity | 6.29 | 1.02 | 7.60 | 3.38 |
| Precipitation | 39.55 | 1.64 | 20.74 | 4.99 |

†Variables with mean weights ≥ 5% were considered as significant contributors to the occurrence of human infections.

“NS”: these variables were excluded from the final model due to small BRT weights (< 2.0%).

**Figure legends to supplementary figures**

**Figure S1. Monthly cumulative numbers of human H7N9 and H5N1 cases reported in mainland China by the end of 2014. Red and blue bars respectively represent monthly cumulative number of human H7N9 and H5N1 cases according to their onset dates.**

**
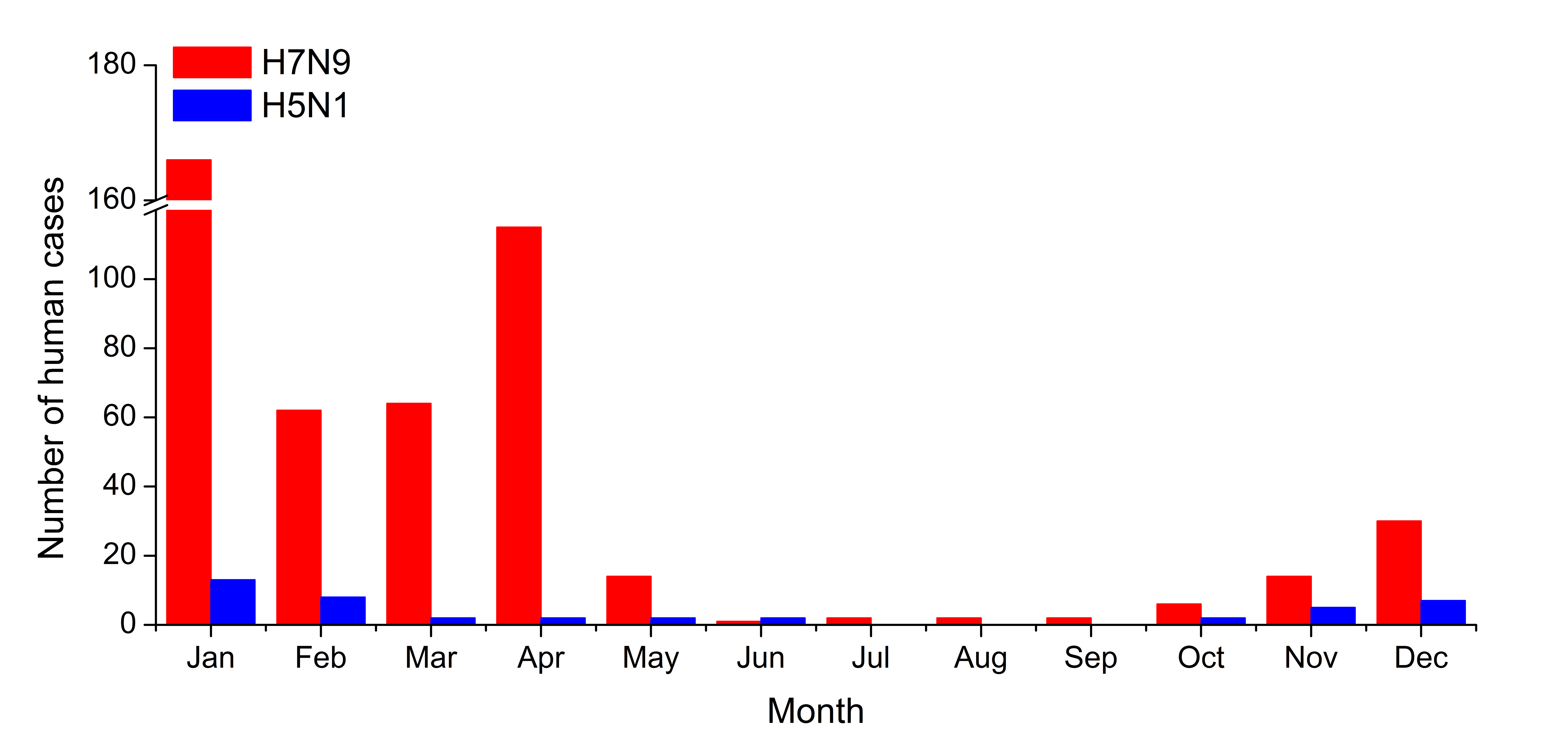
**

**Figure S2. Marginal relationship between predictors and the risk of human infection with avian influenza A (H7N9) based on the BRT model**: (A) number of live poultry markets (LPM), (B) density of poultry (PD_1), (C) density of human population (PD_2), (D) percentage coverage of shrub, (E) percentage coverage of built-up land, (F) temperature (TP), (G) relative humidity (RH), and (H) precipitation (PRE).


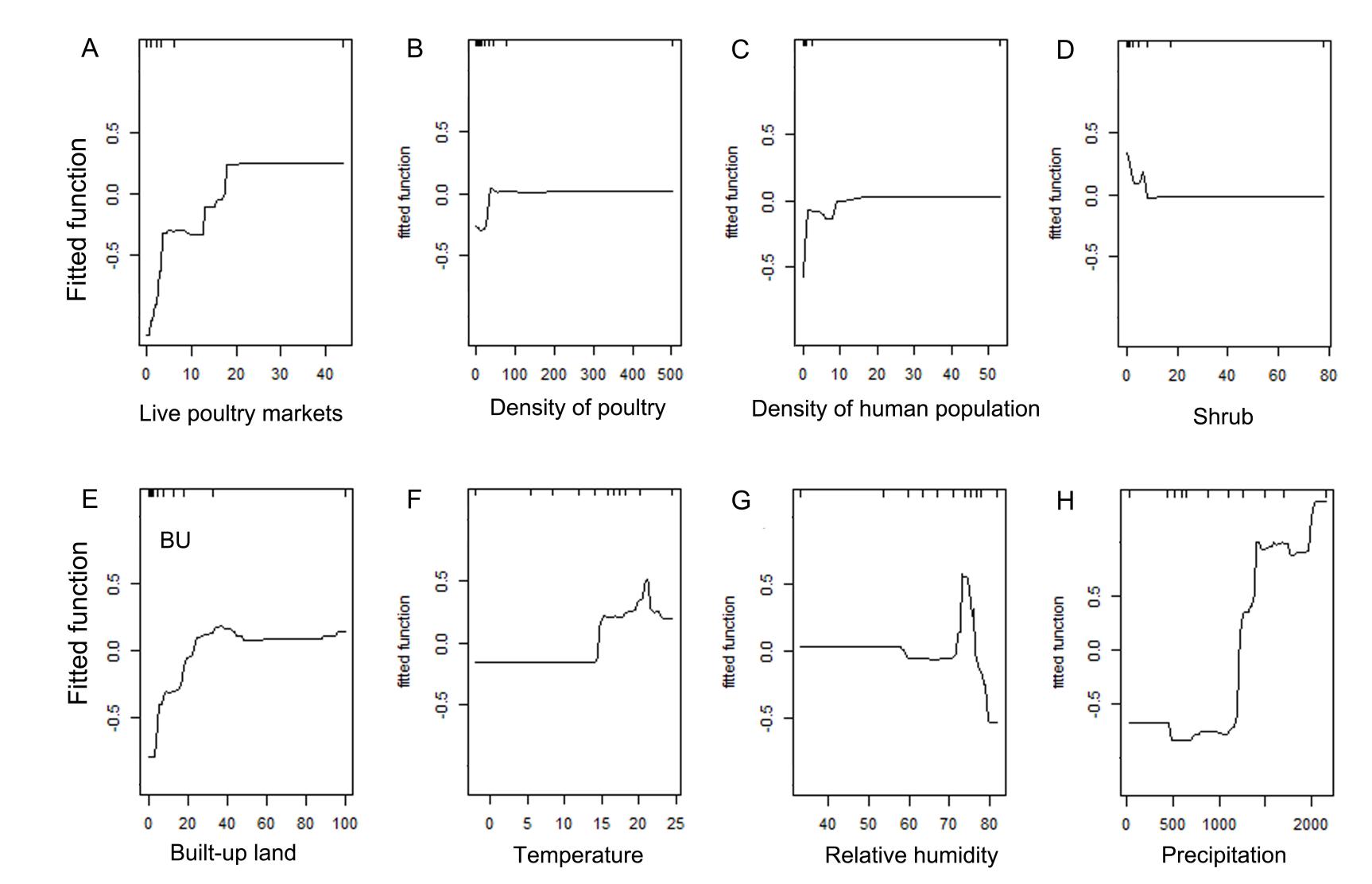


**Figure S3. Marginal relationship between predictors and the risk of human infection with avian influenza A (H5N1) based on the BRT model:** (A) number of live poultry markets (LPM), (B) density of human population (PD_2), (C), percentage coverage of forest (FS), (D) percentage coverage of built-up land, (E) percentage coverage of waterbody (WB), (F) relative humidity (RH), and (G) precipitation (PRE).


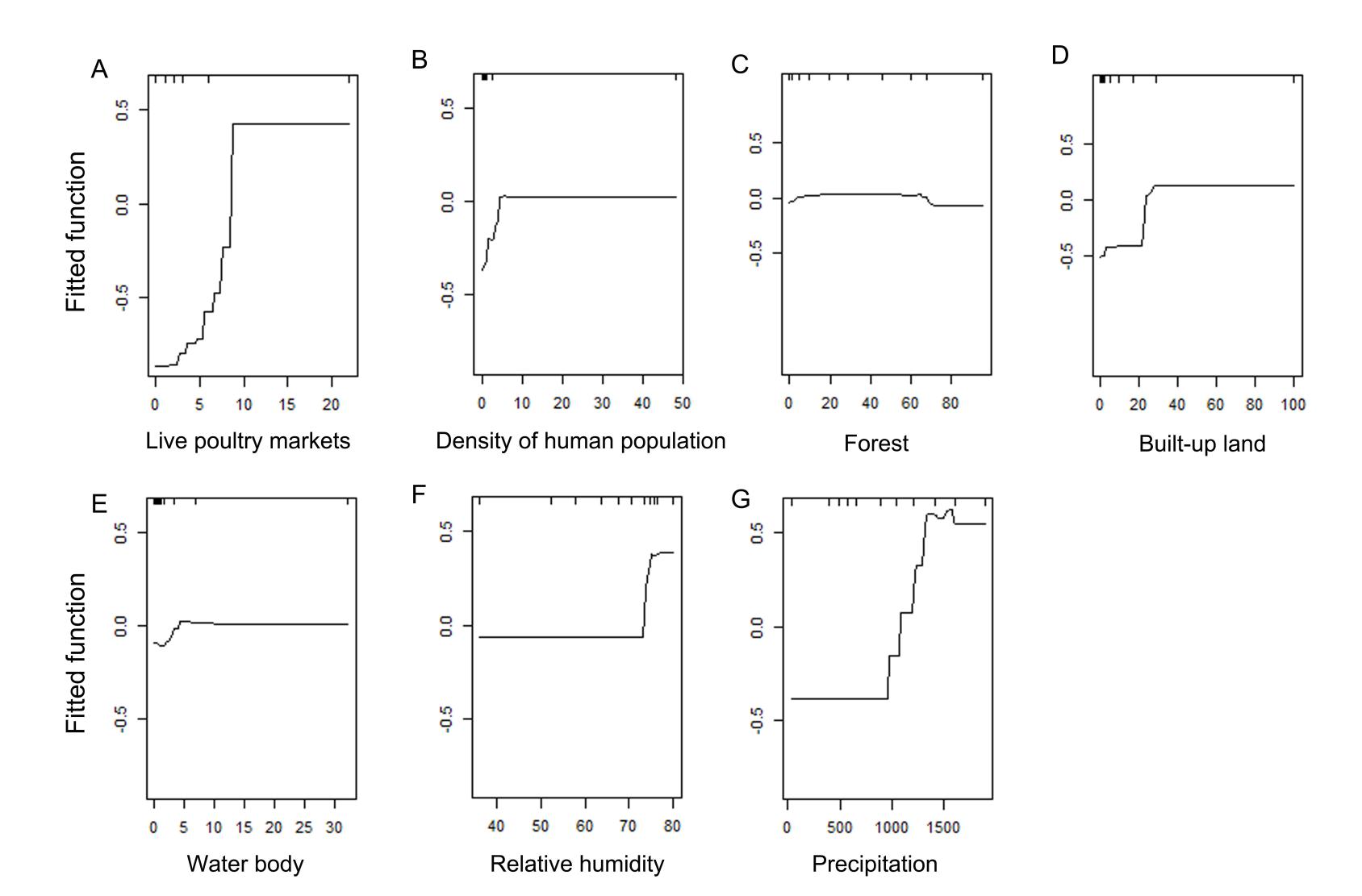


**Figure S4. Receiver operating characteristic (ROC) curves of predicted risk for human infection with (A) avian influenza A (H7N9) and (B) avian influenza A (H5N1).** ROC curves for the 50 resampled datasets are colored in grey. The mean ROC curves over the 50 resamples are colored in black for the training set and either red (H7N9) or blue (H5N1) for the validation set.


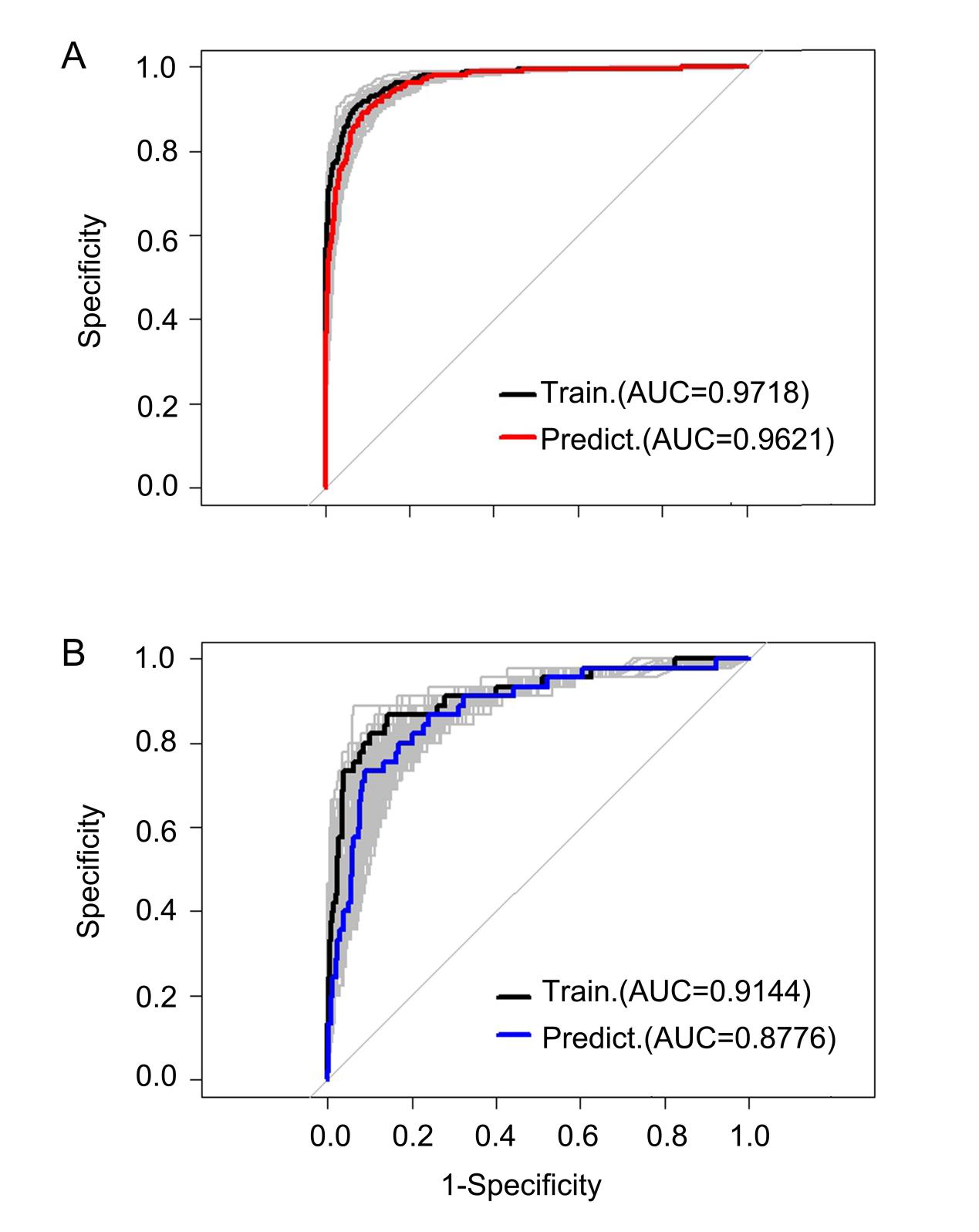

Supplement: Supplementary Information [file srep18610-s1.doc]
